# The Entrepreneur’s Psychological Capital, Creative Innovation Behavior, and Enterprise Performance

**DOI:** 10.3389/fpsyg.2020.01651

**Published:** 2020-07-24

**Authors:** Qianying Gao, Cisheng Wu, Linchuan Wang, Xuyang Zhao

**Affiliations:** School of Management, Hefei University of Technology, Hefei, China

**Keywords:** entrepreneur psychological capital, creative innovation behavior, enterprise performance, structural equation model, multiple regression model

## Abstract

In order to analyze the relationship between entrepreneur psychological capital, creative innovation behavior, and enterprise performance based on the actual situation of Chinese enterprises and provide a theoretical basis for the application of entrepreneur psychological capital in enterprise innovation and performance development, in this study, 536 entrepreneurs from 517 enterprises in different fields in Anhui region were selected, and a questionnaire survey on the psychological capital of entrepreneurs, creative innovation behaviors, and corporate performance was conducted. A hypothesis model of the relationship between entrepreneur’s psychological capital, creative innovation behavior, and enterprise performance was constructed. The correlation between entrepreneur’s psychological capital, creative innovation behavior, and enterprise performance and the intermediation of creative innovation behavior were analyzed using multiple-regression model and structural equation model. The results show that there is a significant positive correlation between dimensions of self-efficacy (regression coefficient = 0.682, *p* = 0.000), toughness (regression coefficient = 0.526, *p* = 0.000), and enterprise performance; there is a significant positive correlation between the dimensions of optimism (regression coefficient = 0.471, *p* = 0.003), hope (regression coefficient = 0.590, *p* = 0.006), and enterprise performance; there is a significant positive correlation between entrepreneurs’ technological innovation behavior (regression coefficient = 0.506, *p* = 0.000), business innovation behavior (regression coefficient = 0.562, *p* = 0.000), and enterprise performance; there is a significant positive correlation between entrepreneurial relationship acquisition behavior (regression coefficient = 0.632, *p* = 0.004) and enterprise performance. Taking entrepreneurs’ creative innovation behavior as the intermediary variable, the authors conclude that the dimensions of entrepreneurs’ self-efficacy, hope, optimism, toughness, and the standardized path coefficient of enterprise performance are significantly reduced; through the analysis of structural equation model, it is found that the fitting index of the model of entrepreneur’s psychological capital, creative innovation behavior, and enterprise performance meets the fitting standard, which shows that both the psychological capital and the creative innovation behavior of entrepreneurs can promote the improvement of enterprise performance. Entrepreneur’s creative innovation behavior plays an intermediary effect in the positive influence of entrepreneur’s psychological capital on enterprise performance.

## Introduction

Psychological capital is a psychological resource that can play a positive role in promoting oneself, development, and the growth of enterprise performance, and it is a positive psychological state that individuals show in their work growth (Bouzari and Karatepe, 2020; Gray et al., 2020). Psychological capital consists of four dimensions, namely, self-efficacy, hope, optimism, and toughness. In recent years, it has become the research object of many scholars in positive organizational behavior (Funken et al., 2020; Ma et al., 2020; Sadq et al., 2020). Through analysis on the online questionnaire and structural equation model completed by 215 science and engineering students in Indonesia, Mahfud et al. (2020) found that the relationship between psychological capital dimensions and entrepreneurial attitude orientation and entrepreneurial intention has a positive partial mediating effect. Kusumawardani et al. (2020) analyzed the entrepreneurial psychology of 352 female entrepreneurs in the Bali tourism industry using the theoretical framework of planned behavior and structural equation model, and found that psychological capital and entrepreneurship education indirectly affected entrepreneurial intention through the two intermediary variables of personal attitude and perceived behavior control. With the advent of the era of economic globalization, many enterprises are facing severe tests. As an important factor for the future development of companies, entrepreneurs also play a very important role in the improvement of corporate performance (Li et al., 2016; Ahmed et al., 2020). As a result, the research on the psychological capital of entrepreneurs is beneficial to the long-term development of enterprises.

Creative innovation has always been a kind of breakthrough thinking pursued by people. For enterprises, the power of creative innovation is the long-term guarantee for the improvement of enterprise performance (Rego et al., 2016, 2017). Since the end of the 20th century, China’s economic and social transformation, entrepreneurship, and innovation economy have become the strategic focus of future development, and also the focus of many scholars. Domi et al. (2020) found in their survey of 211 Albanian tourism SMEs that innovation and innovation behaviors play an intermediary role in the relationship between enterprise synergy and performance. Kurniawan et al. (2020) conducted a questionnaire survey on 150 enterprise personnel in the creative industry and found that there was a significant positive correlation between knowledge sharing and absorptive ability and creative and innovative ability (*p* < 0.05). The creative industry develops better by increasing knowledge sharing, absorptive capacity, and innovation capacity. At present, the mainstream understanding of creative innovation behavior is to regard creative capital as an abstract concept of creativity, and the research mainly focuses on countries and regions and other macro levels, with little involvement in entrepreneurs and individuals (Deligianni et al., 2019; He and Hui, 2020). Therefore, the research on entrepreneurs’ creative innovation behaviors is beneficial to the development of enterprises.

To sum up, the current research on the relationship between entrepreneur psychological capital, creative capital, and organizational performance is too macroscopic, and there are few reports on individual entrepreneurs. Based on this, 536 entrepreneurs from 517 enterprises in different fields in the Anhui region were taken as research objects, and a hypothesis model of the relationship between entrepreneur psychological capital, creative innovation behavior, and enterprise performance was constructed. The relationship between entrepreneur’s psychological capital, creative innovation behavior, and enterprise performance was evaluated using multiple-regression model and structural equation model (Yu et al., 2019).

## Literature Review

As the fourth largest capital in entrepreneurship, the psychological capital of entrepreneurs can directly affect every link in the process of entrepreneurship, which has attracted the attention of many scholars. Baron et al. (2016) proposed that people who were attracted, selected, but persisted in starting a business may have a high bearing capacity, and psychological capital was positively correlated with subjective well-being based on the principle of attract-select-loss. Anglin et al. (2018) expanded the entrepreneurship literature to include positive psychological capital—the level of psychological resources of a person or organization, including hope, optimism, resilience, and confidence—as a significant signal of crowdfunding. It turned out that using positive psychological capital language can improve the performance of crowdfunding. They also looked at 1726 Kickstarter campaigns and found that entrepreneurs who delivered positive psychological capital had excellent fundraising performance. Enterprises need to create benefits to distribute benefits, so enterprise performance is also the most concerned issue in the process of entrepreneurship, and the role of entrepreneurs’ own psychological capital on enterprise performance has attracted the attention of many scholars. Digan et al. (2019) conducted a sample survey of 369 female entrepreneurs of small and medium-sized enterprises in Gujarat, western India, and found that female entrepreneurs can cope with the challenges of self-employment through psychological capital, so as to obtain more corporate benefits from empowerment. Baluku et al. (2018) studied the influence of psychological capital and autonomy on entrepreneurial performance using the self-determination theory and psychological capital literature. It was found that entrepreneurs were satisfied with the influence of psychological capital, autonomy, and their interaction with commitment and that psychological capital had significant influence on training and entrepreneurial performance. Probst et al. (2017) studied whether the “psychological capital” of high-level structural leadership composed of hope, self-efficacy, resilience, and optimism can ease the relationship between job insecurity and performance of employees. The results showed that leaders with high level of psychological capital can reduce employees’ sense of insecurity and improve organizational performance. Edwards and Rowe (2019) constructed a framework for the relationship between psychological capital, employees’ mental health, and corporate performance and tested the role of this structure in employees’ mental health and job satisfaction. The results showed that the psychological capital of leaders can be used as a partial intermediary or a complete intermediary to increase the job satisfaction and mental health of employees, so as to improve the development of enterprise performance. To sum up, the current analysis on the effect of entrepreneur psychological capital on enterprise performance is a hot topic. An in-depth discussion on the relationship between the psychological capital of entrepreneurs, creative innovation behavior, and enterprise performance was explored to provide a theoretical basis for the practical application of psychological capital.

## Methodology

### Research Subjects

In this study, 536 entrepreneurs from 517 enterprises in different fields in Anhui region were selected as research objects. A total of 615 questionnaires were issued, and 579 copies were responded, with a response rate of 94.15%. After careful examination and elimination of invalid data, there were 536 valid questionnaires, with a valid response rate of 87.15%.

The basic characteristics of the enterprise were selected as shown in Table 1. Among the enterprises, private enterprises are the largest, accounting for 54.16%. The Internet enterprises in the industry are the largest, accounting for 39.65%. In terms of company compensation, the number of non-high-paying enterprises is significantly higher than that of high-paying enterprises, accounting for 53.77%. In terms of the number of employees, there are more small enterprises with employees from 1 to 200, accounting for 61.32%.

**TABLE 1:** Basic information of all tested enterprises.

**Enterprises variables**	**Classification**	**Number of the samples**	**Proportion**
Type of the	Private	280	54.16%
enterprise	Foreign capital	84	16.25%
	Sino-foreign joint	59	11.41%
	Other types	94	18.18%
Industry	Internet	205	39.65%
	Real estate	125	24.18%
	Services	134	25.92%
	Other industries	53	10.25%
Pay	High-paying	156	30.17%
	Non-high-paying	278	53.77%
	Not clear	83	16.06%
Number of the	1–200	317	61.32%
employees	201–900	126	24.37%
	More than 900	74	14.31%

The basic characteristics of entrepreneurs are shown in Table 2. In terms of age, entrepreneurs aged 30–40 years are the most, accounting for 37.87%; in terms of educational background, entrepreneurs with bachelor’s degree have the highest level (46.64%); in terms of gender, male entrepreneurs are significantly more than female entrepreneurs, accounting for 63.25%.

**TABLE 2:** Basic information of selected entrepreneurs.

**Entrepreneurs**	**Classification**	**Number of the samples**	**Proportion**
Age	No more than 30	76	14.18%
	30–40	203	37.87%
	40–50	148	27.61%
	More than 50	109	20.34%
Educational	Senior high school and below	56	10.45%
background	Junior college	126	23.51%
	Bachelor degree	250	46.64%
	Master’s degree and above	104	19.40%
Gender	Males	339	63.25%
	Females	197	36.75%

### Research Hypotheses

Enterprise performance is the result of the interaction and matching between the internal data distribution and the external environment, which is influenced by many factors. However, there is no uniform standard for the measurement of enterprise performance in previous studies, and it lacks of official nature. Therefore, a single enterprise performance dimension is proposed and the following hypotheses are put forward.

#### Entrepreneur Psychological Capital and Enterprise Performance

F1: Psychological capital of entrepreneurs has a positive effect on enterprise performance.

F11: Entrepreneurs hope that the dimension has a positive impact on enterprise performance.

F12: The dimension of entrepreneur self-efficacy has a positive influence on enterprise performance.

F13: The entrepreneurial optimism dimension has a positive impact on enterprise performance.

F14: The dimension of entrepreneur toughness has a positive impact on enterprise performance.

#### Entrepreneurial Creative Innovation Behavior and Organizational Learning Ability

F2: Entrepreneurs’ creative innovation behavior has a positive impact on enterprise performance.

F21: Entrepreneurs’ technological innovation has a positive impact on enterprise performance.

F22: The creative innovation behavior of entrepreneurs’ business model has a positive impact on enterprise performance.

F23: The acquisition of entrepreneurial relationship ability has a positive effect on enterprise performance.

#### Entrepreneur Psychological Capital, Creative Innovation Behavior, and Enterprise Performance

F3: Entrepreneur’s creative innovation behavior plays an intermediary effect in the influence of entrepreneur’s psychological capital on enterprise performance.

F31: The technological innovation of entrepreneurs plays a mediating role in the influence of entrepreneur’s psychological capital on enterprise performance.

F32: The innovation behavior of entrepreneur’s business model plays an intermediary effect in the influence of entrepreneur’s psychological capital on enterprise performance.

F33: The behavior of acquiring entrepreneurial relationship ability plays an intermediary effect in the influence of entrepreneurial psychological capital on enterprise performance.

### The Hypothesis Model of the Relationship Between Entrepreneurial Psychological Capital, Creative Innovation Behavior, and Enterprise Performance

**FIGURE 1:**
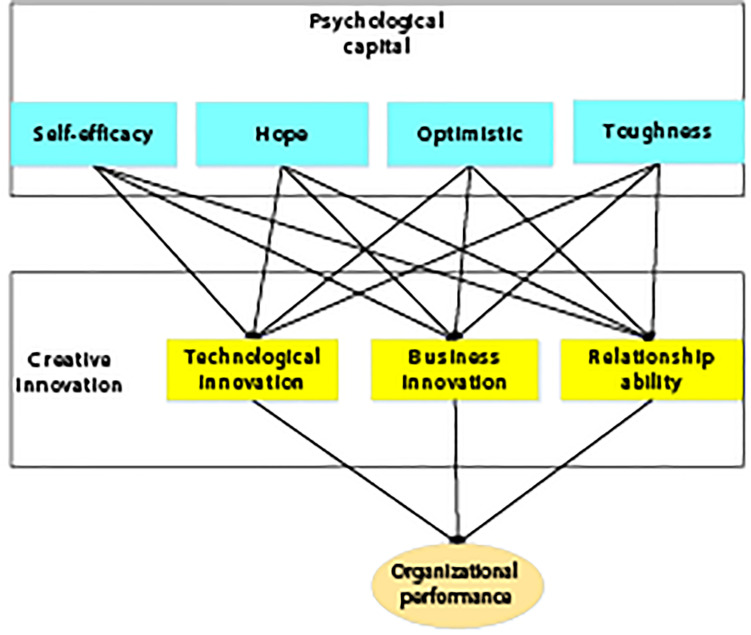
The relationship model of entrepreneur’s psychological capital, creative innovation behavior, and enterprise performance.

In the empirical study, the relationship model of entrepreneur psychological capital, creative innovation behavior, and enterprise performance as shown in Figure 1 can be constructed based on the above hypothesis. The independent variables of this study are entrepreneur psychological capital, including self-efficacy, optimism, hope, and toughness. The intermediary variables are entrepreneur’s creative innovation behavior, including entrepreneur’s technological innovation behavior, entrepreneur’s business model innovation behavior, and entrepreneur’s relationship ability acquisition behavior. The dependent variable is enterprise performance. Based on the current entrepreneurial environment, the psychological capital of entrepreneurs can positively affect the performance of enterprises through creative innovation behaviors.

### Questionnaire Design

In empirical research, questionnaire survey is a common research method used by scholars, which can easily and quickly obtain a lot of required data and has a certain degree of reliability. Therefore, according to the theoretical hypothesis proposed in this study and the relationship model of entrepreneur’s psychological capital, creative innovation behavior, and enterprise performance, the variables to be measured are identified as entrepreneur’s psychological capital, enterprise performance, and entrepreneur’s creative innovation behavior. The scale tools are entrepreneur psychological capital scale, enterprise performance scale, and entrepreneur creative innovation behavior scale.

#### The Entrepreneur Psychological Capital Scale

As shown in Table 3, the questionnaire includes four dimensions: self-efficacy, hope, optimism, and toughness. Self-efficacy consists of six measurement items. The hope dimension includes six measurement items, the optimism dimension includes six measurement items, and the toughness dimension includes eight measurement items. Richter’s five points are used for each item: 1 for strongly disagree, 2 for disagree, 3 for neither agree nor disagree, 4 for agree, and 5 for strongly agree. The internal reliabilities of the four dimensions of self-efficacy, hope, optimism, and toughness and the overall scale are 0.81, 0.79, 0.86, and 0.89, respectively (Laguía et al., 2019).

**TABLE 3:** The entrepreneur psychological capital scale.

**Variables**	**Measurement items**
Self-efficacy	I believe I can solve complicated problems
sense	I believe that I can express and do my job well
	I believe I can make a contribution to the future development of the company
	I believe that I can set the future development goals for the company
	I believe that I can communicate and coordinate the relationship with the outside world
	I believe I can deliver effective information in a timely manner
Hope dimension	I can come up with many solutions when I am faced with difficulties at work
	I can throw myself into my work
	I believe there are many solutions to any problem
	I think I can succeed in my job
	I am confident of achieving and exceeding my goals
	I am achieving the work goals I set for myself
Optimism	I have a positive attitude
dimension	Sometimes I work hard but I still make mistakes
	I always look on the bright side of my work
	I am optimistic about the future development of my work
	All the work runs counter to my ideas
	At work, I always believe that “behind the dark is the light”
Toughness	I will often become depressed and distracted
dimension	In the face of work, I will try every means to solve the problem
	I believe I can do it alone
	In my work, I go all out regardless of my emotions
	I’m getting closer to my goal
	I am calm under pressure
	I’m experienced enough and believe I can handle anything
	I’m full of energy every day

#### The Enterprise Performance Scale

As shown in Table 4, considering that there is no unified standard for enterprise performance, the following table was developed based on previous literature, and the scale adopted seven indicators for evaluation. Richter’s five points were used for each item: 1 for strongly disagree, 2 for disagree, 3 for neither agree nor disagree, 4 for agree, and 5 for strongly agree (Liu et al., 2018).

**TABLE 4:** The enterprise performance scale.

**Variables**	**Measurement items**
Enterprise	The return on total assets is slightly higher than its peers
performance	The return on equity is slightly higher than that of its peers
	The company’s total sales growth is slightly higher than that of its peers
	The competitiveness of the company in the same industry
	Number of patented products
	The position of new products in the industry
	Financial trouble not encountered ever

#### Entrepreneur Creative Innovation Behavior Scale

As shown in Table 5, the scale includes three dimensions: entrepreneur’s technological innovation, entrepreneur’s business innovation, and entrepreneur’s relationship acquisition behavior. Entrepreneur’s technological innovation behavior includes five measurement items, entrepreneur’s business innovation behavior includes three measurement items, and entrepreneur’s relationship acquisition behavior includes four measurement items. Richter’s five points were used for each item: 1 for strongly disagree, 2 for disagree, 3 for neither agree nor disagree, 4 for agree, and 5 for strongly agree (Roopsing and Nokphromphanao, 2018). The internal consistency reliabilities of the three dimensions of entrepreneur’s technological innovation, business innovation, and relationship acquisition behavior and the overall scale are 0.86, 0.83, and 0.91, respectively.

**TABLE 5:** Entrepreneur creative innovation behavior scale.

**Variables**	**Measurement items**
Technological innovation	The management considers the company’s ability to learn to be our competitive advantage
of entrepreneurs	The company regards learning as one of the core values for future development and improvement
	The company sees learning as an investment rather than a cost
	The enterprise sees active learning as a necessary quality for future survival
	The enterprise does not attach importance to the learning of employees
Entrepreneurial	I focus on business model innovation
business creativity and innovation	I encourage my employees, customers, and others to provide ideas and suggestions for business model innovation
	I see continuous innovation in business models as an important part of the organization
Entrepreneurial relationship acquisition	Our company and partners often exchange relevant information and experience
behavior	In cooperation with others, both parties are willing to provide helpful information
	Our company and our partners are willing to provide our own proprietary information
	We will inform each other of events or changes that may affect each other

### Analysis Method

SPSS19.0 was used to process the data in this study. The counting data were expressed as a percentage (%). The relationship between entrepreneur’s psychological capital and enterprise performance, the relationship between entrepreneur’s creative innovation behavior and enterprise performance, and the intermediary effect of entrepreneur’s creative innovation behavior on enterprise performance were investigated using AMOS 24.0 software. Besides, Spearman correlation was used to analyze the correlation between each dimension of entrepreneur’s psychological capital, creative innovation behavior, and enterprise performance. Afterward, the *t*-test was used for comparison of different demographic variables in the psychological capital group. *p* < 0.05 indicates that the difference is statistically significant.

## Results

### Difference Analysis of Different Demographic Variables

As shown in Table 6, in terms of gender, the psychological capital toughness of male is significantly higher than that of female (*p* < 0.05). In terms of education background, self-efficacy of entrepreneurs with bachelor’s degree, master’s degree, or above is significantly higher than that of junior college, senior high school or below (*p* < 0.05). In terms of age, the optimism dimension of entrepreneurs under 30 years of age is significantly higher than that of entrepreneurs over 30 years old (*p* < 0.05). The hope dimension of psychological capital of entrepreneurs under the age of 0 is significantly higher than that over the age of 40 (*p* < 0.05).

**TABLE 6:** Analysis on the difference of psychological capital of different gender, age, and educational background.

**Variables**		**Self-efficacy**	**Optimism**	**Toughness**	**Hope**
Gender	Male	24.58	26.75	34.58a	25.08
	Female	25.12	26.36	29.59b	25.96
Educational background	Senior high school and below	23.19a	25.99	32.51	25.61
	Junior college	24.07a	26.16	32.74	25.17
	Bachelor’s degree	26.84b	26.31	33.16	25.24
	Master’s degree and above	27.01b	26.07	32.35	26.02
Age	No more than 30	24.93	27.32a	32.45	26.32a
	30–40	25.08	25.33b	31.94	26.17a
	40–50	25.19	25.91b	32.84	24.91b
	More than 50	24.60	25.61b	32.80	24.86b

*Different letters a and b indicate that pairwise comparison in the group has statistical significance (*p* < 0.05).*

### Spearman Correlation Analysis of Entrepreneur Psychological Capital, Creative Innovation Behavior, and Enterprise Performance

Spearman correlation was first used to analyze whether there was a significant correlation between the psychological capital of entrepreneurs, creative innovation behavior, and enterprise performance. As shown in Table 7, the dimensions of entrepreneurs’ self-efficacy and toughness are significantly correlated with enterprise performance (*p* < 0.001); the dimensions of entrepreneurs’ hope and optimism are significantly correlated with enterprise performance (*p* < 0.05). Among them, the correlation between self-efficacy and enterprise performance is the strongest (*r* = 0.801).

**TABLE 7:** Spearman correlation analysis between each dimension of entrepreneur psychological capital and enterprise performance.

**Entrepreneur psychological capital**	**Self-efficacy**	**Hope**	**Optimism**	**Toughness**
Entrepreneur psychological capital *r*	0.801	0.653	0.710	0.758
*P*	0.000	0.006	0.003	0.000

As shown in Table 8, there is a very significant correlation between entrepreneurs’ technological innovation behavior and business innovation behavior and enterprise performance (*p* < 0.001); there is a significant correlation between entrepreneurial relationship acquisition behavior and enterprise performance (*p* < 0.05). Among them, the correlation between technological innovation and enterprise performance is the strongest (*r* = 0.859).

**TABLE 8:** Spearman correlation analysis between the dimensions of entrepreneurs’ creative innovation behavior and enterprise performance.

**Entrepreneur psychological capital**	**Technological innovation**	**Business creativity and innovation**	**Relationship acquisition behavior**
Enterprise performance *r*	0.859	0.817	0.736
*P*	0.000	0.000	0.004

### Empirical Research on Entrepreneur Psychological Capital and Enterprise Performance

As shown in Table 9, every dimension of entrepreneurs’ psychological capital was taken as the independent variable, and organizational performance, age (under 30 years of age, 0; 30–40 years of age, 1; 40–50 years of age and over 50 years of age, 2; more than 50, 3), educational background (below high school education, 0; junior college, 1; bachelor’s degree, 2; master or above, 3), and gender (male, 1, female, 0) were taken as dependent variables. The regression coefficient of self-efficacy and organizational performance is 0.682, which has a very significant positive correlation (*p* < 0.001). Thus, the hypothesis “F12: entrepreneurs’ self-efficacy dimension has a positive impact on enterprise performance” is true. The regression coefficient between optimism dimension and organizational performance is 0.475, which has a significant positive correlation (*p* < 0.05). Thus, hypothesis F13 “entrepreneur’s optimism dimension has a positive impact on enterprise performance” is true. The regression coefficient between the expected dimension and organizational performance is 0.590, with a significant positive correlation (*p* < 0.05). Thus, the hypothesis “F11: entrepreneurs’ hope dimension has a positive impact on enterprise performance” is true. The regression coefficient between toughness dimension and organizational performance is 0.526, showing a very significant positive correlation (*p* < 0.001). Thus, hypothesis “F14: entrepreneur toughness dimension has a positive impact on enterprise performance” is true. The regression coefficient between educational background and enterprise performance is 0.543, showing a significant positive correlation (*p* < 0.05).

**TABLE 9:** Regression analysis of entrepreneur psychological capital and enterprise performance.

**Models**	***T*-value**	**Regression coefficient**	***p***
Self-efficacy	5.617	0.682	0.000
Optimism	4.835	0.471	0.016
Toughness	6.118	0.526	0.000
Hope	5.389	0.590	0.024
Age	1.472	0.217	0.067
Educational background	5.825	0.543	0.013
Gender	1.642	0.271	0.076

The relationship between variables was handled through the analysis of entrepreneur psychology scale and structural equation model. Self-efficacy was expressed by the English abbreviation SE, hope dimension is expressed by HO, optimism dimension was expressed by OP, toughness dimension was expressed by TO, and enterprise performance is expressed by PE.

**FIGURE 2:**
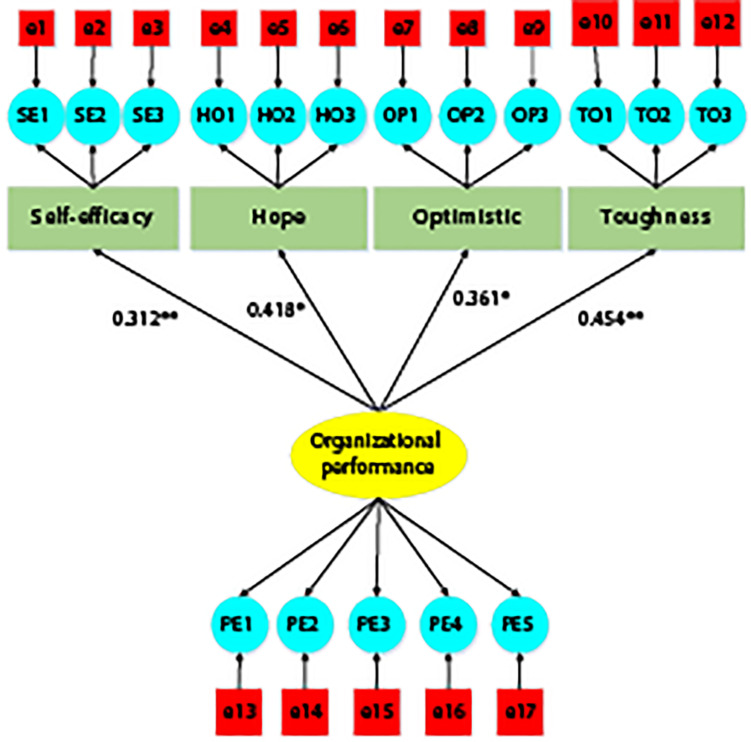
The structural equation path analysis of the relationship model between entrepreneur psychological capital and enterprise performance. Note: * meant significant at *p* < 0.05; ** meant significant at *p* < 0.001.

As shown in Figure 2, self-efficacy, hope dimension, optimism dimension, and toughness dimension are external latent growth variables, and enterprise performance belongs to internal latent growth variables. In addition, there are 17 residual variables. The standardized path coefficient of self-efficacy and enterprise performance is 0.312, the standardized path coefficient of hope dimension and enterprise performance is 0.418, the standardized path coefficient of optimism dimension and enterprise performance is 0.361, and the standardized path coefficient of toughness dimension and enterprise performance is 0.454. Table 10 is the fitting index of the relationship model between entrepreneur psychological capital and enterprise performance. The X^2^ fitting priority index (X^2^/df), the root-mean-square error approximation (RMSEA), the goodness of fitting test (GFI), the normalized fitting index (NFI), the Tucker–Lewis index (TLI), the cross validity test index (ECVI), and the comparison fitting index (CFI) all meet the fitting standard, and the fitting evaluation is ideal.

**TABLE 10:** The fitting index of the relationship model between entrepreneur psychological capital and enterprise performance.

**Test indicator**	**X^2^/df**	**RMSEA**	**GFI**	**NFI**	**TLI**	**ECVI**	**CFI**
Fitting results	2.109	0.052	1.137	1.295	1.070	0.683	1.033
Fitting evaluation	Ideal	Ideal	Ideal	Ideal	Ideal	Ideal	Ideal

### An Empirical Study on Entrepreneurs’ Creative Innovation Behavior and Enterprise Performance

As shown in Table 11, the multiple linear regression analysis was conducted with each dimension of entrepreneurs’ creative innovation behavior as independent variable and organizational performance as dependent variable, age, educational background, and gender as the control variables. The regression coefficient between technological innovation and organizational performance is 0.506, showing a very significant positive correlation (*p* < 0.001). Thus, the hypothesis “F21: entrepreneurs’ technological innovation has a positive impact on enterprise performance” is true. The regression coefficient between business innovation and organizational performance is 0.562, showing a very significant positive correlation (*p* < 0.001). Thus, hypothesis “F22: entrepreneurs’ creative innovation behavior in business model has a positive impact on enterprise performance” is true. The regression coefficient of relationship acquisition ability and organizational performance is 0.632, showing a significant positive correlation (*p* < 0.01). Thus, the hypothesis “F23: the acquisition of entrepreneurial relationship ability has a positive impact on enterprise performance” is established. The regression coefficient between educational background and enterprise performance is 0.496, showing a significant positive correlation (*p* < 0.05).

**TABLE 11:** Regression analysis of entrepreneurs’ creative innovation behavior and enterprise performance.

**Models**	***T*-value**	**Regression coefficient**	***p***
Technological innovation	4.591	0.506	0.000
Business creativity and innovation	5.380	0.562	0.016
Relationship acquisition	5.628	0.632	0.008
Age	3.752	0.362	0.051
Educational background	5.796	0.496	0.011
Gender	2.371	0.301	0.072

**FIGURE 3:**
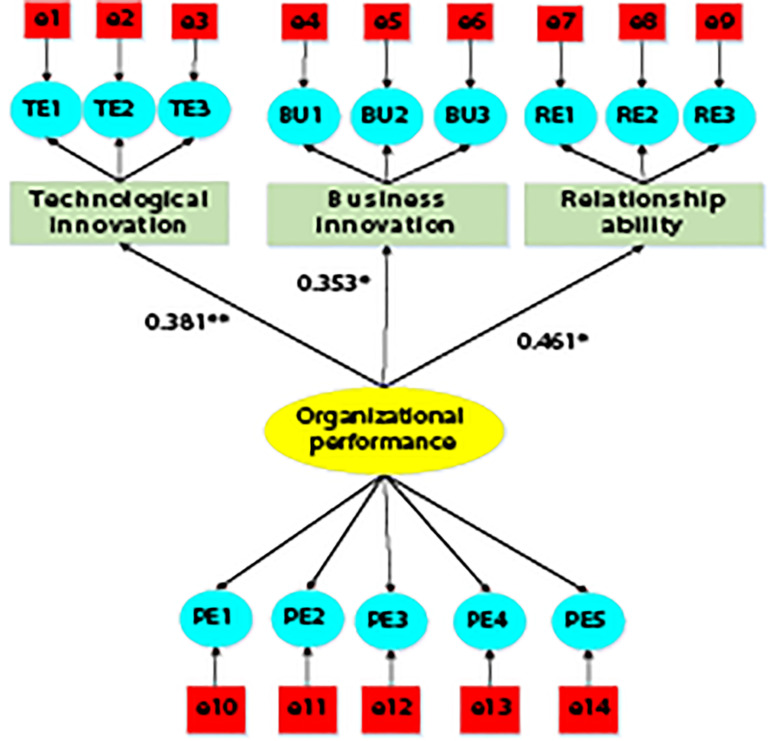
The structural equation path analysis of the relationship model between entrepreneur’s creative innovation behavior and enterprise performance. Note: * meant significant at *p* < 0.05; ** meant significant at *p* < 0.001.

As shown in Figure 3, technical innovation behavior is represented by the English abbreviation TE, business innovation behavior is represented by BU, and entrepreneurial relationship acquisition behavior is represented by RE. Technological innovation behavior, business innovation behavior, and entrepreneur relationship acquisition behavior are extrinsic latent growth variables, and enterprise performance is intrinsic latent growth variables. In addition, there are 14 residual variables. The standardized path coefficient of technological innovation and enterprise performance is 0.381, the standardized path coefficient of business innovation and enterprise performance is 0.353, and the standardized path coefficient of relationship acquisition ability and enterprise performance is 0.461. Table 12 shows the fitting index of the relationship model between entrepreneurs’ creativity and innovation and enterprise performance. The X^2^/df, RMSEA, GFI, NFI, TLI, ECVI, and CFI all meet the fitting standard, and the fitting evaluation is ideal.

**TABLE 12:** The fitting index of the relationship model between entrepreneur’s creative innovation behavior and enterprise performance.

**Test indicator**	**X^2^/df**	**RMSEA**	**GFI**	**NFI**	**TLI**	**ECVI**	**CFI**
Fitting results	1.973	0.059	1.206	1.347	1.255	0.551	1.170
Fitting evaluation	Ideal	Ideal	Ideal	Ideal	Ideal	Ideal	Ideal

### Empirical Research on Entrepreneur’s Psychological Capital, Creative Innovation Behavior, and Enterprise Performance

**FIGURE 4:**
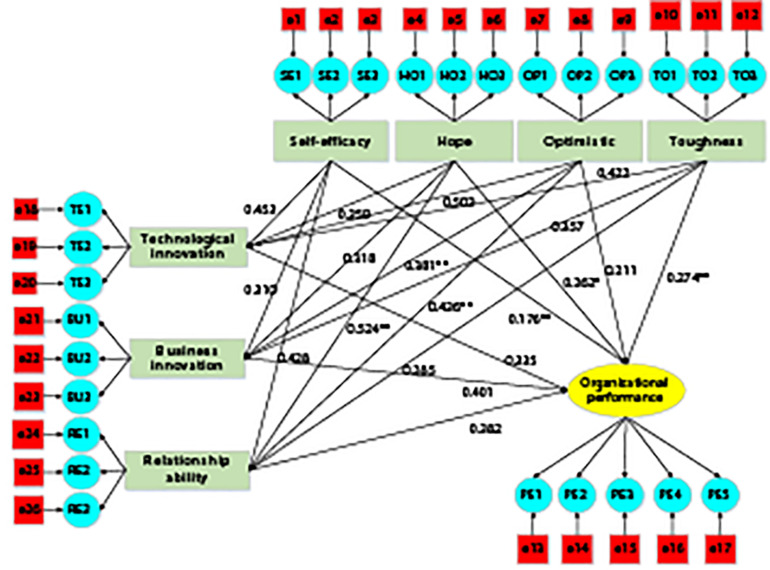
The relationship between entrepreneurial psychological capital, creative innovation behavior, and enterprise performance path. Note: * meant significant at *p* < 0.05; ** meant significant at *p* < 0.001.

As shown in Figure 4, the entrepreneur’s creative innovation behavior was taken as the intermediary variable of the entrepreneur’s psychological capital and enterprise performance, and the structural equation modeling method is applied to analyze its mechanism of action. After the inclusion of intermediary variable entrepreneurs’ creative innovation behavior, the standardized path coefficient has a significant change.

After incorporating entrepreneurial technological innovation, entrepreneurial business innovation, and entrepreneurial relationship to obtain behavioral variables, the standardized path coefficient of entrepreneur self-efficacy and enterprise performance is significantly reduced from 0.312 to 0.176; the standardized path coefficient of the dimension of entrepreneurs’ hope and enterprise performance decreases significantly from 0.418 to 0.262, the standardized path coefficient of the dimension of entrepreneurs’ optimism and enterprise performance decreases significantly from 0.361 to 0.211, and the standardized path coefficient of the dimension of entrepreneurs’ toughness and enterprise performance decreases significantly from 0.454 to 0.274; the standardized path coefficient of entrepreneurs’ technological innovation and enterprise performance is 0.335; and the standardized path coefficient of entrepreneurs’ business innovation and enterprise performance is 0.401. The standardized path coefficient of entrepreneurial relationship acquisition behavior and enterprise performance is 0.382. Thus, the hypothesis “F3: entrepreneur’s creative innovation behavior is the intermediary variable of entrepreneur’s psychological capital affecting enterprise performance, and entrepreneur’s psychological capital influences enterprise performance through entrepreneur’s creative innovation behavior” is true.

## Discussion

Entrepreneurs are the beneficial implementers of entrepreneurial plans and activities, which determine the success of entrepreneurship. How to develop the psychological capital of entrepreneurs in the current competitive social background is an urgent problem to be solved. Therefore, the questionnaire survey method was combined with structural equation model analysis method to study 517 enterprises in different fields in Anhui region. First, it is found that the dimension of psychological capital toughness of males is significantly higher than that of females (*p* < 0.05), which is the same as the research results of Digan et al. (2019), suggesting that male entrepreneurs are better able to cope with stress and difficulties. The self-efficacy of entrepreneurs with bachelor’s degree, master’s degree, or above is significantly higher than that of junior college, senior high school, or below (*p* < 0.05), which is similar to the research results of Anokye and Asumeng (2019), suggesting that highly educated entrepreneurs have a higher degree of confidence in achieving their goals. The optimism dimension of entrepreneurs under 30 years of age is significantly higher than that of entrepreneurs over 30 years of age, and the hope dimension of entrepreneurs under 40 years of age is significantly higher than that of entrepreneurs over 40 years of age (*p* < 0.05). This may be because entrepreneurs under the age of 30 are more unmarried, have fewer worries about starting a business, and are more optimistic, while entrepreneurs under the age of 40 are regarded as the golden age of entrepreneurs, full of hope for the future (Nordin et al., 2019). The dimensions of self-efficacy and toughness are significantly positively correlated with organizational performance (*p* < 0.001), while the dimensions of optimism and hope are significantly positively correlated with organizational performance (*p* < 0.05). This is consistent with the research results of Wang et al. (2018), suggesting that all dimensions of entrepreneur psychological capital can promote the improvement of enterprise performance, but the dimensions of self-efficacy and toughness have higher positive influence than the dimensions of hope and optimism. This may be because the dimension of optimism and hope is the self-confident expression of entrepreneurs themselves, which can promote entrepreneurs to carry out innovative activities but cannot guarantee the quality of activities (Lucky, 2018). According to the analysis of the structural equation model, it is found that the fitting indexes of the relationship model between the entrepreneur’s psychological capital and enterprise performance all meet the fitting criteria, which indicates that the model is supported. Thus, the hypothesis “F1: entrepreneur psychological capital has a positive impact on enterprise performance” is true.

In order to maintain the competitive advantage of enterprises in the same industry, entrepreneurs need to take risks, actively launch new products and services, and explore new markets, which requires entrepreneurs to have efficient creative innovation behaviors (Asad et al., 2018; Qian et al., 2018). In this study, it is found that entrepreneurs’ technological creative innovation behavior, business creative innovation behavior, and organizational performance have extremely significant positive correlation (*p* < 0.001), while the standardized regression coefficient of entrepreneurial relationship acquisition behavior and organizational performance is 0.632, showing a significant positive correlation (*p* < 0.05). This is consistent with the research results of Subramanian et al. (2019), which shows that each dimension of entrepreneur’s creative innovation behavior has a positive promoting effect on enterprise performance, but entrepreneur’s technological creative innovation behavior and business creative innovation behavior have higher influence than entrepreneur’s relationship acquisition behavior. This may be because although entrepreneurs can make use of divergent thinking to obtain a broader knowledge space in relation degree and complete the integration of various resources, they cannot directly promote innovation activities (Obschonka et al., 2018). In addition, the structural equation model analysis found that the fitting index of the model of the relationship between entrepreneurs’ creative innovation behavior and enterprise performance met the adaptation criteria, indicating that the model was supported. Thus, the hypothesis “F2: entrepreneurs’ creative innovation behavior has a positive impact on enterprise performance” is true.

In addition, after the entrepreneur’s creative innovation behavior was regarded as the intermediary variable of the entrepreneur’s psychological capital and enterprise performance, and the structural equation model was used to analyze and find that after the entrepreneur’s creative innovation behavior variable was included, the dimensions of entrepreneurs’ self-efficacy, hope, optimism, and toughness and the standardized path coefficient of enterprise performance were significantly reduced. This is basically consistent with the research results of Shin et al. (2018), suggesting that the psychological capital of entrepreneurs can exert a positive effect on the performance of enterprises through the intermediary of entrepreneurs’ creative innovation behaviors (Chen, 2019; Gonçalves de Lima et al., 2020). This is because the psychological capital of entrepreneurs can stimulate the divergent thinking of entrepreneurs, enhance the ability of technological and business creativity and innovation, explore new profit models, and finally continuously improve the level of enterprise performance (Pierre Johnson et al., 2019; Yan et al., 2019).

## Conclusion

By establishing the relationship model of entrepreneur’s psychological capital, creative innovation behavior, and enterprise performance path, the authors analyzed the influence of various dimensions of entrepreneur’s psychological capital, creative innovation behavior on enterprise performance, and the mediation of entrepreneur’s creative innovation behavior on enterprise performance, providing a theoretical basis for the improvement of enterprise performance in the future (Shen et al., 2019). However, due to the limitations of time and economic capacity, there is no clear standard for the measurement dimension of enterprise performance. The innovative performance was used to represent enterprise performance, which had certain ambiguity. Subsequently, the enterprise performance can be divided into dimensions to make the evaluation more accurate. In a word, both entrepreneurial psychological capital and creative innovation can promote the improvement of enterprise performance (Zheng and Liu, 2020). The psychological capital of entrepreneurs can exert a positive effect on the performance of enterprises through the intermediary of entrepreneurs’ creative innovation behaviors.

## Data Availability Statement

All datasets generated for this study are included in the article/supplementary material.

## Ethics Statement

The studies involving human participants were reviewed and approved by the Hefei University of Technology Ethics Committee. The patients/participants provided their written informed consent to participate in this study.

## Author Contributions

QG: writing—original draft preparation. CW: writing—review and editing. LW: methodology. XZ: validation. All authors contributed to the article and approved the submitted version.

## Conflict of Interest

The authors declare that the research was conducted in the absence of any commercial or financial relationships that could be construed as a potential conflict of interest.
